# Supplementary material for: Torularhodin Alleviates Hepatic Dyslipidemia and Inflammations in High-Fat Diet-Induced Obese Mice via PPARα Signaling Pathway
Source: Molecules. 2022 Sep 27;27(19):6398. doi: 10.3390/molecules27196398 (PMC9572851; doi:10.3390/molecules27196398)
Supplement: Supplementary file 1 [file molecules-27-06398-s001.zip › molecules-1896724-supplementary.pdf]

Table S1. The up-regulated proteins in HFD-T group vs HFD group.

Genes	FC*	Protein Name	Access number	Physiological function
Ndufs8	1.27	NADH dehydrogenase [ubiquinone] iron-sulfur protein 8, mitochondrial	NDUS8_MOUSE	Catalyzes NADH to NAD+ by electron transfer in the respiratory chain (β-oxidation increases NAD/NADH ratio)
Gk	1.36	Glycerol kinase	GLPK_MOUSE	Increased glycerol kinase activity indicate increased lipid oxidation (release of glycerol)
Apoa2	1.53	Apolipoprotein A-II	APOA2_MOUSE	A component of high density lipoproteins (HDL).
Ugt2a3	1.53	UDP-glucuronosyltransferase 2A3	UD2A3_MOUSE	Make lipophilic substrates with increased water solubility favoring excretion
Vamp8	1.55	Vesicle-associated membrane protein 8	VAMP8_MOUSE	VAMP8 is a SNARE protein involved in autophagy
Sec61b	1.58	Protein transport protein Sec61 subunit beta	SC61B_MOUSE	Mediates transport of signal peptide-containing precursor polypeptides across the ER
Gulo	1.61	L-gulonolactone oxidase	GGLO_MOUSE	An old gene to synthesize vitamin C of L-ascorbate.
Eci1	1.61	Enoyl-CoA delta isomerase 1, mitochondrial	ECI1_MOUSE	Involved in the fatty acid beta-oxidation
Asgr1	1.69	Asialoglycoprotein receptor 1	ASGR1_MOUSE	Endocytosis of plasma glycoproteins，Asparagine N-linked glycosylation
Emc1	1.69	ER membrane protein complex subunit 1	EMC1_MOUSE	Indirectly involved in many cellular processes by regulating the insertion of various proteins into the membrane.
Eci2	1.70	Enoyl-CoA delta isomerase 2	ECI2_MOUSE	Involved in the fatty acid beta-oxidation
Tmem30a	1.74	Cell cycle control protein 50A	CC50A_MOUSE	Transport of aminophospholipids, beneficial to phospholipid balance (PS&PE) in the membrane
Ephx1	1.75	Epoxide hydrolase 1	HYEP_MOUSE	Detoxification of toxic metabolites such as epoxide-containing fatty acids.
Rps10	1.75	40S ribosomal protein S10	RS10_MOUSE	Component of the 40S ribosomal subunit
Ddc	1.76	Aromatic-L-amino-acid decarboxylase	DDC_MOUSE	Neuromessenger of dopamine serotonin synthesis
Pck1	1.87	Phosphoenolpyruvate carboxykinase, cytosolic [GTP]	PCKGC_MOUSE	Rate-limiting step in gluconeogenesis, related to glucose and lipid balance.
Apoa4	2.28	Apolipoprotein A-IV	APOA4_MOUSE	Apoa-IV is a structural component of HDL
Fabp5	2.41	Fatty acid-binding protein 5	FABP5_MOUSE	Fatty acid binding protein family (FABP). participating in fatty acid uptake and metabolism
Cyp7a1	3.20	Cytochrome P450 7A1	CP7A1_MOUSE	Involved in cholesterol catabolic process.
Cpt1a	3.85	Carnitine O-palmitoyltransferase 1, liver isoform	CPT1A_MOUSE	Involved in carnitine metabolic process; promoting fatty acid β-oxidation
Acaa1a	1.51	3-ketoacyl-CoA thiolase A, peroxisomal	THIKA_MOUSE	Involved in fatty acid beta-oxidation in peroxisomes
Acaa1b	2.71		THIKB_MOUSE	Fatty acid metabolic process，promote fatty acid β-oxidation in the liver

	3-ketoacyl-CoA thiolase B, peroxisomal

*FC：　fold changes

Table S2. Down-regulated proteins in HFD-T group.

Gene	FC*	Protein Name	Access Number	Physiological Function
Plin2	0.08	Perilipin-2	PLIN2_MOUSE	Coating lipid droplet， associated with adipocyte differentiation, adipose tissue development and maintenance
Slc27a5	0.36	Bile acyl-CoA synthetase	S27A5_MOUSE	Hepatic fatty acid uptake and bile acid reconjugation and recycling
Slc27a4	0.38	Long-chain fatty acid transport protein 4 （FATP4）	S27A4_MOUSE	Principal fatty acid transporter in small intestinal enterocytes
Fabp1	0.42	Fatty acid-binding protein, liver	FABPL_MOUSE	Lipoprotein-mediated cholesterol uptake in hepatocytes
Fabp2	0.42	Fatty acid-binding protein,intestinal	FABPI_MOUSE	Long-chain fatty acid transport, and intestinal lipid absorption
Asl	0.45	Argininosuccinate lyase	ARLY_MOUSE	L-arginine biosynthesis and urea cycle
Sorbs1	0.57	Sorbin and SH3 domain-containing protein 1	SRBS1_MOUSE	Sertoli-Sertoli Cell Junction Dynamics pathway, involved in insulin-stimulated glucose transport, and formation of actin stress fibers and focal adhesions
Vnn1	0.57	Pantetheinase	VNN1_MOUSE	Recycling pantothenic acid (vitamin B5) and releasing cysteamine and involved in cell proliferation
Ppm1k	0.57	Protein phosphatase 1K, mitochondrial	PPM1K_MOUSE	Regulates the mitochondrial permeability transition pore and promote branched-chain amino acid catabolism
Glod4	0.60	Glyoxalase domain-containing protein 4	GLOD4_MOUSE	Metalloenzyme in the glycolytic pathway involved in the detoxification of reactive methylglyoxal into d-lactate using glutathione (GSH) as cofactor
Me1	0.61	NADP-dependent malic enzyme	MAOX_MOUSE	Energy providers (through generating NADPH) for fatty acid synthesis
Plin5	0.63	Perilipin-5	PLIN5_MOUSE	Coat lipid droplet, maintain lipid droplet homeostasis by regulating both the lipid storage and fatty acid oxidation
Plin4	0.73	Perilipin-4	PLIN4_MOUSE	coat intracellular lipid storage droplets

*FC: fold changes vs HFD group.

Table S3. Inflammation-associated proteins in HFD-T group. .

Gene	FC*	Protein	Access No.	Function
Gsdmd	2.16	Gasdermin-D	GSDMD_MOUSE	Immune defense and pyroptosis
Fas	1.63	Tumor necrosis factor receptor superfamily member 6	TNR6_MOUSE	TNF-receptor involved in cell apoptosis
Bax	1.56	Apoptosis regulator BAX	BAX_MOUSE	Programmed cell death
Icam1	1.55	Intercellular adhesion molecule 1	ICAM1_MOUSE	Resolution of inflammatory conditions.
Ocln	1.41	Occludin	OCLN_MOUSE	Cytokine can reduced its expression
Gstp1	1.33	Glutathione S-transferase P 1	GSTP1_MOUSE	Suppress LPS induced pro-inflammatory factors
Faf1	1.27	FAS-associated factor 1	FAF1_MOUSE	Apoptosis by interacting with the Fas receptor
Optn	0.79	Optineurin	OPTN_MOUSE	Negative regulation of NF-kappaB signaling
Ptk2b	0.62	Protein-tyrosine kinase 2-beta	FAK2_MOUSE	Macrophage and sites of inflammation
Fadd	0.60	FAS-associated death domain protein	FADD_MOUSE	Involved in inflammatory effects
Mif	0.54	Macrophage migration inhibitory factor	MIF_MOUSE	A lymphokine involved in inflammation
Casp3	0.43	Caspase-3	CASP3_MOUSE	Apoptosis, necrosis and inflammation.
Yap1	0.41	Transcriptional coactivator YAP1	YAP1_MOUSE	Transcription of the inflammatory cytokine IL-1β
Lrp1	1.37	Low-density lipoprotein receptor-related protein 1	LRP1_MOUSE	Suppressor expression of inflammatory mediators
Apex1	1.91	DNA endonuclease	APEX1_MOUSE	Promotes inflammation by orc hestrating the NF-κB
Rock1	1.47	Rho-associated protein kinase 1	ROCK1_MOUSE	Suppressor of inflammatory cell migration （negative）
Manf	1.61	Mesencephalic astrocyte-derived neurotrophic factor	MANF_MOUSE	Inhibiting Inflammation by negative transcriptional effect on NF-kB Pathway
Stat3	1.41	Signal transducer and activator of transcription 3	STAT3_MOUSE	Anti-inflammatory （regulate gene expression）
Dnm1l	0.66	Dynamin-1-like protein	DNM1L_MOUS	Promote inflammation by AKT/IKK/NFKBIA/NF-κB NAD immunometabolism，

Nampt	0.63	Nicotinamide	E	promoting gut inflammation
		phosphoribosyltrans	NAMPT_MOUS	
		ferase	E	Anti-inflammation
Insr	1.99	Insulin receptor		Detoxification of toxic
Ephx1	1.75	Epoxide hydrolase 1	INSR_MOUSE	metabolites such as epoxide-
			HYEP_MOUSE	containing fatty acids

*Fold changes compared to the HFD group.
